# Cryptococcose neuroméningée et tuberculose osseuse chez un immunocompétent: un cas

**DOI:** 10.11604/pamj.2015.20.109.6055

**Published:** 2015-02-06

**Authors:** Mariam Gbané-Koné, Boubacar Ouali, Estelle Mègne, Mohamed Diomandé, Abidou Kawalé Coulibaly, Edmond Eti, N'zué Marcel Kouakou

**Affiliations:** 1 Service de Rhumatologie CHU de Cocody, Abidjan, Cote d'Ivoire

**Keywords:** Méningite, cryptococcose neuroméningée, VIH, immunocompétent, tuberculose, Meningitis, Cryptococcal meningitis, HIV, immunocompetent, tuberculosis

## Abstract

Les auteurs rapportent un cas de cryptococcose neuroméningée (CNM) chez un patient de 39 ans, non infecté par le VIH suivi pour une tuberculose de hanche. Le tableau clinique était celui d'une méningite subaiguë. Le diagnostic a été possible grâce à la mise en évidence de cryptocoques dans le LCR. L’évolution a été satisfaisante sous fluconazole.

## Introduction

La cryptococcose neuroméningée (CNM) est une affection mycosique rare, survenant le plus souvent chez des sujets ayant une infection par le VIH [1, 2]. Nous rapportons le cas d'un patient de 39 ans, sans facteur apparent d'immunodépression suivi pour une ostéoarthrite tuberculeuse et qui à deux semaines du traitement antituberculeux, présenta un tableau de méningite aigue fébrile rattachée à une cryptococcose neuroméningée. Le diagnostic fut établi par la mise en évidence de capsules de cryptocoques dans le liquide céphalo-rachidien.

## Patient et observation

**Figure 1:**
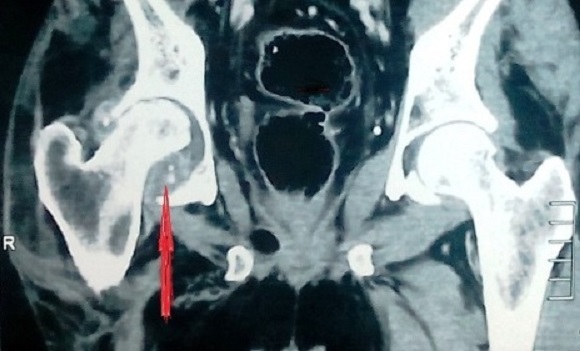
(TDM du bassin) ostéoarthrite coxofémorale avec des abcès contenant des calcifications

**Figure 2:**
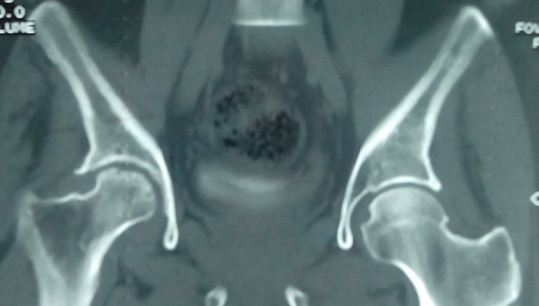
(TDM du bassin) à 12 mois de traitement antituberculeux, ostéosclérose cicatricielle

Un homme de 39 ans, a été hospitalisé pour une coxopathie droite inflammatoire, d'installation progressive, évoluant depuis 20 mois. Cette symptomatologie évoluait dans un contexte de fébricule vespérale, une asthénie, une anorexie et un discret amaigrissement. Dans ses antécédents, on retrouvait un zona thoracique et une notion de contage tuberculeux franc (sa mère a souffert d'une tuberculose pulmonaire), il n’était pas diabétique, ni drépanocytaire. L'examen physique a mis en évidence, des signes de coxopathie droite avec boiterie à la marche, un clino-statisme et une amyotrophie du quadriceps. A la palpation on retrouvait une douleur au pli inguinal et du grand trochanter, sans tuméfaction. La flexion et les rotations étaient limitées. L'examen des autres articulations était normal de même que l'examen général. A la radiographie du bassin de face, on notait un pincement global de l'interligne articulaire, des érosions avec destruction de la tête fémorale et de la cotyle. La TDM du bassin a confirmé l'ostéo-arthrite de la hanche droite avec des abcès contenant des calcifications (Figure 1). La radiographie pulmonaire et l’échographie abdominale étaient normales. A la biologie, on notait une hyperleucocytose à 13 000 /mm^3^ à prédominance neutrophile, une discrète anémie hypochrome microcytaire à 12,1g/dl, un discret syndrome inflammatoire avec (VS à 28 mm à H1, CRP normale). L'IDR à la tuberculine était positive à 10mm. Les hémocultures étaient stériles de même que le tubage gastrique. Le bilan rénal et hépatique était normal de même que la glycémie. La sérologie rétrovirale était négative de même que les marqueurs de l'hépatite virale (VHB et VHC). Une ponction scannoguidée des abcès de la hanche a été réalisée et elle a mis en évidence des BAAR. Le diagnostic de coxalgie a été retenu et le patient mis sous traitement antituberculeux. Une traction de la hanche dans le plan du lit a été réalisée. Au 15^è^ jour du traitement, le tableau s'est brutalement compliqué, le patient se plaignait de céphalées intenses en casque; associées à des vomissements en jet, une photophobie avec reprise de la fièvre à 39 voire 40° (alors qu'initialement on avait un train subfébrile). L'examen physique retrouvait un syndrome méningé franc, sans déficit sensitivomoteur et une altération brutale de l’état général (7kgs en 1 semaine). Une localisation cérébrale de la tuberculose a été évoquée et nous avons réalisé en urgence une ponction lombaire (après un fond d’œil normal). La PL a ramené un liquide clair dont l'analyse chimique a montré une hyper protéinorachie à 1,75g/L, une hypoglucorachie à 0,37g/L, la cytologie du LCR montrait 90 éléments à prédominance lymphocytaire. La recherche de mycobactéries, tant à l'examen direct qu'en culture sur milieux spéciaux était négative. L'examen systématique du LCR à la coloration à l'encre de chine a mis en évidence des cryptocoques. Le diagnostic de CNM a été retenu. En plus du traitement antituberculeux, le patient a été mis sous Fluconazole à la dose de 1200mg/j pendant 14 jours puis 800 mg/j pendant 8 semaines. L’évolution a été satisfaisante avec une apyrexie franche au bout de 3 jours du traitement, une régression des céphalées et du syndrome méningé. Le patient a été revu en consultation régulièrement pendant 12 mois et demeurait asymptomatique. Au plan articulaire, on a également eu une régression de la douleur, la marche était redevenue possible, le traitement de la cryptococcose a duré 5 mois, celui de la tuberculose, un an. La TDM du bassin de contrôle montrait une ostéosclérose cicatricielle sans abcès(Figure 2). Avec un recul de 16 mois l’évolution est toujours favorable et la sérologie rétrovirale était toujours négative

## Discussion

La cryptococcose est une infection grave due à une levure encapsulée, Cryptococcus neoformans. Il s'agit d'un champignon ubiquitaire isolé du sol et des fientes de pigeons [3]. La particularité de cette observation, c'est la présentation clinique trompeuse, alors que tout nous orientait vers une tuberculose neuroméningée (tuberculose osseuse en cours de traitement, méningite à liquide clair à prédominance lymphocytaire), l'analyse du LCR concluait plutôt à une CNM. Notre patient étant immunocompétent, c'est un diagnostic auquel nous n'avons pas pensé d'emblée. En effet, dans la littérature, la CNM est décrite comme étant la mycose systémique la plus fréquente au cours du sida [4]. Elle constitue la deuxième affection opportuniste au cours du sida [4, 5] et elle affecte surtout les malades à un stade avancé de l'infection par le virus de l'immunodéficience humaine. D'autres affections peuvent cependant favoriser son apparition, ce sont les hémopathies malignes, les collagénoses, les traitements immunosuppresseurs, la tuberculose, la grossesse, les insuffisances hépatique et rénale graves et le diabète [2]. Cliniquement, la CNM se manifeste généralement par un tableau de méningo-encéphalite subaigüe dont le diagnostic repose sur l’étude du LCR [2, 4, 6]. La chimie du LCR révèle une hypercytorachie modérée, prédominant aux lymphocytes, une hyperprotéinorachie modérée, une hypoglycorachie et une hypochlorurachie [2]. Trois possibilités pour le diagnostic de certitude: soit à l'examen direct après une coloration à l'encre de chine, ou en culture sur milieu de Sabouraud, ou par la recherche d'antigène cryptococcique [1, 2, 4, 6]. Le deuxième intérêt de ce dossier, c'est la bonne évolution de la CNM sous Flucazole avec une guérison sans séquelles neurologiques, quand on sait le fort taux de mortalité de cette affection, en effet dans une étude réalisée au Cameroun [6] sur la CNM au cours du VIH, le taux de mortalité s’élevait à 42,2%, Au Burkina [4], cette mortalité était plus élevée et précoce (80% des patients décédaient avant 15 jours). La mortalité de l'atteinte neuro-méningée est de 100% en l'absence de traitement, elle est de 25 à 30% chez les sujets traités, avec plus d'un tiers de séquelles neurologiques [2]. Le traitement classique de la CNM reste l'association amphotéricine B-flucytosine, quelque soit la le statut sérologique [1, 2, 6], cependant plusieurs études ont montré l'efficacité du Fluconazole en monothérapie et à forte dose [6–8]. Chez ce patient, nous pensons que la tuberculose osseuse a été le facteur favorisant de cette grave affection.

## Conclusion

Cette observation rappelle qu'une cryptococcose neuroméningée peut survenir chez des sujets sans facteur apparent d'immunodépression avec une présentation clinique peu spécifique. Cela souligne l'intérêt de la recherche systématique des cryptocoques devant toute méningite et ce, quelque soit le statut sérologique du patient.
